# Difficult Dentition

**Published:** 1881-04

**Authors:** Edward N. Buckingham


### ARTICLE VI.

### *Difficult Dentition.*

BY EDWARD M. BUCKINGHAM, M. D.

Read before the Boston Society for Medical Observation, December 1, 1879.

The subject of the following paper was presented to me by my having the care of a case which, while in its main features not unusual, presented certain points of interest, one of which, I think, has not been much discussed, and another, though a matter on which every one has an opinion, depends for its understanding upon certain physiological facts, which I have not seen grouped together.

A boy of one year, having two teeth, and never having fully recovered his strength since being sick with cholera infantum, six months before, had been ill for some days. His diet was a thick mixture of Ridge's food and water, with an equal amount of milk. He had diarrhœa, meal and cheese being mixed with the fæces, vomiting and cough, for which examination of the chest gave no explanation. Milk and water, equal parts, were ordered, with the addition of an alkali. Ridge's food was omitted.

Second Day. No vomiting after the second meal, and diarrhœa ceased. Tympanitic abdomen and much rigidity of legs. Fingers in mouth and swollen gum over the middle superior incisors. I cut the gum.

Third to eighth day. Skin of extremities red, shining, swollen and tender, but without fever. These appearances ended in a sharp line toward the trunk, advanced over the whole surface, faded without desquamation, and completed their course in three days. Once only, a little cheese in the dejections. Appetite improved, and in eight days he was well, but the teeth had not appeared.

Thirteen days later I learned that directions had been followed for two days only, during which time he was well; then the proportion of milk had been increased, and cheese had appeared in the fæces. In consequence the alkali had been abandoned and Ridge's food substituted. Matters not improving, milk had been repeatedly reduced, so that for two days the diet had been merely Ridge's food and water. During these days the dejections had been mostly meal, the child had been crying, and the fingers had become rigid. The same diet was ordered as before. He stopped crying on getting it, and had a comfortable night.

The next morning the gums were rather swollen, nowhere much so, and he was cross and restless. At midnight he began to breathe with a grunt, and early in the following morning the head was hot, fontanelle throbbing violently, pupils contracted and not reaching, lips livid, jaws set tight, and abdomen tympanitic; the gums were swollen, especially at the site of the old cut. He having been in a warm bath without effect, I again cut in the same place, but owing to the contraction of the jaw, or my own clumsiness, without hitting the tooth. There was more bleeding than usual. During the next fifteen minutes the child got his first sleep in twenty-four hours, and the fontanelle ceased throbbing. On waking at the end of that time, he received an injection, evacuating wind and a little fæces of good appearance. About noon, Dr. C. P. Putnam saw him with me, and made some incisions crossing mine. The pupils were then reacting much better than in the morning, but there were fever and rales in the chest. 6.30 P. M. Had taken food, and had a green dejection. Temperature higher; gums more generally swollen and red, and had bled repeatedly. I cut over four teeth of the lower jaw, and he was bathed and oiled. Three hours later, the temperature still rising, I cut two more teeth, thus completing the incisions where the swelling was marked, and he was again bathed. Rigidity continued and an hour after midnight he died.

An autopsy was made fifty-seven hours after death, Dr. Draper being present. The fontanelle was much sunken. The brain was very much injected: not much serum in the ventricles. No pathological changes. Kidneys and liver normal. Lungs engorged in lower lobes, but crepitating. Calvarium cartilaginous. Heart, bladder and intestines not examined.

Although the case is, excepting for the continued bleeding, by no means uncommon, it illustrates certain points in the treatment of children, notably, gum cutting, the use of starch and the difference in physiological age. Twice during my attendance the gums were cut. On the first occasion there seems to have been no effect, the improvement being remote, and after much trouble. On the second, the effect, though only temporary, seemed for the moment to be brilliant, the more or less comatose condition of several hours disappearing at once in sleep, the violent throbbing of the fontanelle diminishing from the first, and the pupils, which were greatly contracted and altogether irresponsive to light, being, when next examined, much better in both respects. It remains to be proved that the relief was consequent upon the incisions, but the connection was certainly interesting. That this one cut was not the only thing wanting is shown by the death, which may or may not have been owing, in part, to the delay in completing the incisions. It should be stated, however, that the swelling of the seat of the latter incisions was but slight until late in the day.

I have cut into swollen gums on twenty-one different occasions. Of course, this number is altogether too small to be regarded as affording anything more than a slight contribution to the study of the subject, and yet the study of these cases may not be without interest. Of them, one may, I think, be fairly subtracted, because the indications for it were not fairly marked, and the failure of what had been done as giving a bare chance of relief ought not to be compared with cases in which there was positive expectation of it.

Of the remaining twenty occasions, in one, the first incision in the reported case, the cut entirely failed. In one, the group of cuts on the last day of this patient's life, the incisions, at first promised well, finally utterly failed. The remaining eighteen were all followed rather closely by a certain amount of improvement.

In a few of these cases I have delayed cutting, hoping to reach the desired end by regulating diet, and this may somewhat alter the value of the statistics. It is, however, my impression that in those cases in which I have not been allowed to use the gum lancet, improvement has been less rapid. In another group of these eighteen cases, gum cutting has constituted the whole treatment, and in three at least of these last, the fretful child of the previous twenty-four hours has recovered its temper and spirits within an hour, and had an improved appetite for its next meal, or the next but one.

Two cases seem worthy of special mention. One, a little girl, still walking and speaking with difficulty, apparently remembering the relief given on a similar occasion with another tooth, asked me to cut her gum. That this was a request, and not an exclamation, was made clear by her coming toward me as she spoke. The other, whose gum I had cut at fourteen months, during the absence of his physician, who had afterward repeated the cut, came to his father, when suffering from a later tooth, with a knife from the dinner table, and a request for the operation. These two cases I allude to because in them the relief to suffering must have been considerable and immediate.

The case we have been considering has for its one unusual, not unique, feature bleeding from the gum, and the cause of this bleeding seems worth investigating. There was an unusually great, but by no means very great loss of blood at once, and during the afternoons the cut repeatedly oozed, though in the opinion of the nurse to no great extent. This had the effect, when I decided in the early evening to extend the cuts, to make me do it half at a time, in order that I might not have too great a bleeding surface to do with in case of accident, there being still two molar teeth over which I then thought the gum needed cutting, which was done a few hours later. Now the one positive thing shown by the autopsy was that the brain was overcharged with blood, and it has since suggested itself to me that anything that would have helped to restore the natural condition of things there, and anything that would have diminished the uneasiness in the gum, and so lesson the grinding, would have lessoned the probability of bleeding; that is, that there would have been less chance of bleeding had the incisions been finished at once. I offer this as a possible explanation of this case of bleeding, not of all cases, and to ask for criticism.

Then the question of the diet of this child is an interesting one. I hope we shall learn from some one present if teething never causes disturbance in nervous children who are properly fed and cared for. But it is certain that illfed children have the reputation of suffering most. Still we hear of so many children who eat all sorts of things and are well, that the question of starch digestion is interesting, and especially so in view of the fact that many children get it in small quantities as an adjunct to milk, and are reported as thriving. In consequence, I have searched for reports of experiments bearing on the subject.

Although the saliva, even of very young children, has been reported to have the power of turning starch into sugar, yet the food must be in contact with it but a very short time before passing into the stomach, and in the stomach of adults at least diastatic action seems to be hindered.

Prospero Sonsino, of Florence, found that the fresh infusion of the pancreas of five young sucklings, dogs, rabbits and a cat, had no diastatic action upon starch even after a long time, while the similar infusion made from adults transformed it almost immediately. He made similar experiments on the enteric juice, though with less satisfactory results, but some of them appeared to show him that the intestine transformed starch at an earlier age than the pancrease. Sonsino himself raises the question how far we may reason here from analogy.

Korowin, as the children's clinic at St. Petersburg, made examinations that, in connection with those of Sonsino, appear to be of more value than either alone, inasmuch as both reached somewhat the same result, the one through children who had died sick, the other through animals who had died well. Korowin tried to turn starch into sugar by means of the pancreas of children who died of different diseases, mostly of the chest and intestines, and who were examined at different periods after death. He found no diastatic action during the first month, a little during the second, enough for quantitative analysis at the end of the third, and the quality fully developed at the end of the first year. His report, which is valuable, would, I think, be more so if he had divided his intestinal and non-intestinal diseases into two groups.

I am indebted to the note-book of Dr. G. M. Garland for an account of examinations by Cr. Hans Wegschreider, who succeeded in converting starch into sugar by means of the fresh fæces of sucklings. He sometimes got a reaction in an hour, a strong one in twenty-four hours. These experiments agree with those of Sonsino on the infant intestine. The value, however, of Sonsino's experiments on the intestine is diminished by the observation of Pasceutin, that the mucous membrane of other organs in dogs has diastatic power.

Besides these experiments in cooking starch in secretions and in infusions of organs, there remain those made directly upon the intestinal contents. of young starch-fed children. In twelve autopsies made by M. Guillot, of children dead from different diseases, he found that both the small and the large intestine contained starch, as shown by a deep blue with tincture of iodine. I believe that there is a fuller account of these autopsies than I have been able to find, but that the children were quite young appears probable from their being quoted by Valleix in an article based on observations on the newly born. Zweifel examined the body of a seven days' child, born at term, and fed exclusively on Nestle's *kindermehl.* The contents of the stomach were slimy and swollen, containing sugar and especially starch, which though its swelling had pressed the stomach out of place. The large intestine contained only unaltered starch. It would seem from this report that the child must have been liberally supplied with it, but it is to be observed that the remaining parts of the diet were disposed of. One analysis of the kindermehl shows sugar, fat, salts, etc., and thirty per cent. starch and dextrine. Other analyses give little starch.

Of course, in any experiments made at an autopsy, we are dealing with the organs of the sick, and it is always a fair question how much the sickness has altered the conditions. While we may have to wait a long time for the combination of a group of early autopsies in starch-fed babies, dead from violence, yet meanwhile we can examine the fæces of living children.

Prospero Sonsino made ten such examinations, as follows: (1.) Sick child of three months. Nestle's kindermehl. Starch shown in fæces by iodine and microscope. (2.) Enlargement of glands of neck, atrophy and diarrhœa at six months. Diet lactose. No starch in fæces. (3.) Well except for club-foot. Three months. Lactose. Strong blue color with iodine. (4.) Condition not stated. Fifteen months. Beef tea and lactose. No starch in fæces. (5.) Convalescent from pneumonia. Five years. Starchy food. A trace of starch in fæces. (6.) Empyma and rickets. Three years. Milk and starch. Fæces affords strong evidence of starch. (7.) Condition not stated. Three months. Mother's milk and bread. Fæces containing starch. (8.) Condition not stated. Twelve months. Milk and flour. No starch in fæces. (9.) Condition not stated. Age four and one half months. Groats, corn flour and milk. Starch abounds in fæces. (10.) Condition not stated. Ten months. Beef tea, yolk of egg, milk, and twice a day one teaspoon of arrowroot. Fæces contain a little starch.

It is interesting to notice that the pneumonia-convalescent and the patient with empyema and rickets did not digest all their starch, though far beyond the age at which Korowin's examinations would lead us to expect that they would: also that the ten-months' child, health unknown, failed to dispose of two teaspoons of arrowroot, but a mixture of four different articles.

The most valuable cases of the series, as bearing on digestion under ordinary circumstances, are the five months' boy, well but for club-foot, who passed unaltered starch, and three children of twelve to sixteen months, of whom one at least was ill, who digested it.

During the past two weeks I have tried to repeat these experiments, but being unwilling to be the means of making your children eat starch, have but one case to report. This is a perfectly healthy boy of eleven months, having five teeth, and eating Indian and oat meal, bread, cracker and milk. I have repeatedly examined his fæces with iodine, always with a negative result.

It seems to me that in examining fæces for this purpose, either sick children should be rigorously rejected, or at least they should be arranged in a separate group. Such statistics, though less numerous, would be more valuable. It seems to be shown by this group of experiments that while there may be some question as to what is the earliest age at which starch is digested, it certainly is, in some children, in very small amount before the third month, and that as a rule children cannot digest it before the second year, perhaps later, to the same extent that adults can. These results seem to be reached most definitely by the experiments of Korowin. We may get still more definite ideas with increased knowledge of the infant intestine. The examination of recent infant fæces seems also a promising field of inquiry. But we can never arrive at exact results owing to what has been called "the difference of physiological age" in children at the same period after birth,—a difference perfectly well recognized. Compare, for instance, the case with which this paper was begun; a year old child, with two teeth, who passed Ridge's food unaltered through the bowels, with the other boy of eleven months, who had five teeth, and whose fæces gave no reaction with iodine, in spite of bread cracker and meal. It is well known that many physicians are in the habit of ordering starch in small quantity with milk, and getting good results, and it has been shown by Dr. Putnam that starch so given may make more digestible without being itself digested. It seems to be desirable, where this practice is followed, to have it thoroughly understood by the attendants that they are dealing, not with a food, but a drug, and a powerful drug, in order to hinder such abuse from ignorance as in the reported case in which starch was not recommended at all.—*Bost. Med. and Surg. Journal.*

